# Synthesis and CO_2_/N_2_ Separation Performance Analysis of Mixed Matrix Membrane (MMM) Based on Different Bimetallic Metal–Organic Frameworks (Ni-Cu-MOF-74, Ni-Co-MOF-74, and Ni-Zn-MOF-74)

**DOI:** 10.3390/membranes15120385

**Published:** 2025-12-18

**Authors:** Shoaib Ahsan, Muhammad Ahsan, Tayyaba Noor, Sarah Farrukh, Humais Roafi

**Affiliations:** School of Chemical and Materials Engineering (SCME), National University of Sciences and Technology (NUST), Sector H-12, Islamabad 44000, Pakistan; sahsan.phdscme@student.nust.edu.pk (S.A.); tayyaba.noor@scme.nust.edu.pk (T.N.); sarah.farrukh@scme.nust.edu.pk (S.F.); hroafi.che9scme@student.nust.edu.pk (H.R.)

**Keywords:** mixed matrix membranes, metal–organic frameworks, gas separation, carbon capture

## Abstract

Polydimethylsiloxane (PDMS) is commonly used in gas-separation studies because of its high CO_2_ permeability and stable mechanical properties. In this work, mixed matrix membranes (MMMs) were prepared by incorporating the bimetallic MOFs Ni-Cu-MOF-74, Ni-Co-MOF-74, and Ni-Zn-MOF-74 into a PDMS matrix. The membranes were fabricated by solution casting and characterized by SEM, XRD, FT-IR, and BET analyses, which confirmed uniform filler dispersion and the successful incorporation of the MOF-74 structures. Single-gas permeation tests showed clear performance improvements with MOF loading. The best results were obtained for the membrane containing 1 wt.% Ni-Cu-MOF-74, which reached a CO_2_ permeability of 3188.25 Barrer and a CO_2_/N_2_ selectivity of 35.10. The improvement is attributed to the accessible metal sites and high surface area provided by the MOF-74 framework, which enhanced adsorption–diffusion pathways for CO_2_ transport. These results show that PDMS/MOF-74 mixed-matrix membranes are effective for CO_2_/N_2_ separation, with Ni-Cu-MOF-74 achieving the highest performance.

## 1. Introduction

The rapid growth of industrial activity and global economic development has caused a continuous rise in carbon dioxide (CO_2_) emissions, with direct consequences for the climate and for public health. In the early stages of industrialization, most emissions originated from developed countries, but in recent decades, contributions from developing nations have increased sharply. This shift has made international coordination necessary rather than relying on a small group of emitters. An important step in this direction was the Kyoto Protocol, adopted in 1997, which established a framework for limiting greenhouse gas emissions globally [1]. CO_2_ is of particular concern because it enhances the greenhouse effect and contributes directly to atmospheric warming [2]. Among the main greenhouse gases, it stands out for its large emissions and long atmospheric residence time, both of which intensify its environmental and health impacts [3,4]. The combustion of fossil fuels remains the dominant source of anthropogenic CO_2_. Typical flue gas from fossil-fuel-based power plants and industrial processes contains about 12–15% CO_2_ and 70–75% N_2_, along with smaller amounts of other gases [5]. Due to these high emissions levels, effective carbon capture technologies are required to mitigate climate-related risks and reduce environmental burden. In parallel, CO_2_ is increasingly recognized as a valuable carbon source for large-scale chemical production, providing additional incentive to design efficient capture and utilization processes [6]. At present, carbon capture and sequestration is mainly carried out through four technological routes: adsorption, absorption, cryogenic distillation, and membrane-based gas separation, each with its own operating window and separation limitations [7].

Among these, membrane-based gas separation has emerged as a practical option for large-scale CO_2_ capture because it typically requires lower operating costs and less energy and can be integrated with relatively simple process equipment [8,9]. These features make it particularly suitable for industrial use. A wide range of membrane materials has been investigated for gas separation and can be broadly classified as organic or inorganic. Organic membranes are usually dense, non-porous polymeric materials [10], whereas inorganic membranes are based on ceramic and metallic systems, including amorphous silica, zeolites, and palladium alloys [11].

Most membrane materials still present important limitations. Ceramic membranes, for example, tend to develop cracks during fabrication, which reduces their separation performance [12], and their large-scale manufacture remains expensive [13]. Metallic membranes also pose difficulties, including embrittlement, high operating costs, and limited service lifetimes [14]. In contrast, polymeric membranes are widely used because they are easier to fabricate and operate and can offer good selectivity [15,16,17]. However, their relatively low gas permeability results in the well-known permeability–selectivity trade-off described by Robeson’s upper bound [18,19]. Overcoming this trade-off is essential for the development of high-performance membrane systems [20]. One strategy to address this issue is the design of hybrid polymer–inorganic systems, or mixed matrix membranes (MMMs), in which inorganic fillers are dispersed within dense polymer matrices [21,22]. MMMs combine the good processability of polymers with the thermal and chemical stability of inorganic phases. Their enhanced CO_2_ separation performance is largely due to the intrinsic high gas separation capabilities of the inorganic components [23]. Among these, metal–organic frameworks (MOFs) have become especially attractive as fillers for MMM fabrication because they typically offer good compatibility with polymer matrices, very high surface area and porosity, and a strong affinity for CO_2_ [24,25,26,27,28,29]. Recent work using MOFs as fillers in MMMs has reported significant improvements in CO_2_ separation performance. For instance, M.A. Rodrigues et al. [30] incorporated MIL-101(Cr) into a polyurethane matrix and obtained a CO_2_/N_2_ selectivity of 42.4 with a CO_2_ permeability of 83.1 Barrer. In another study, Q. Zhang et al. [31] prepared triptycene-functionalized polyimide MMMs containing ZIF-90, achieving a CO_2_/N_2_ selectivity of 26.18 and a CO_2_ permeability of 12.75 Barrer, surpassing the 2008 Robeson upper bound. More recent work has aimed to alleviate the permeability–selectivity trade-off by creating MOF-based transport pathways within polymer matrices. For example, the use of amino-modified UiO-66 in a polysulfone matrix resulted in high CO_2_ permeability and reasonably high CO_2_/N_2_ selectivity [32]. Likewise, surface-modified UiO-66 incorporated into a Matrimid matrix enhanced permeation properties and produced a modest improvement in CO_2_/N_2_ selectivity [33]. Coating strategies have also been used to improve MOF-based MMMs. For example, MIL-101(Cr) particles coated with polyethyleneimine and dispersed in a sulfonated poly (ether ether ketone) matrix produced membranes whose performance exceeded the 2008 Robeson upper bound [34]. To further enhance the separation properties of MOF-filled MMMs, bimetallic MOFs have been investigated. M. Loloei et al. [35] incorporated a bimetallic Zn-Co ZIF (ZIF-8-67) into a 6FDA-ODA polyimide matrix and reported a maximum CO_2_/CH_4_ selectivity of 45.1 and a CO_2_ permeability of 44.2 Barrer. Compared with MMMs containing monometallic ZIF-8 or ZIF-67, the bimetallic filler provided higher selectivity and permeability. In a related study, X. Du et al. [36] synthesized a bimetallic UiO-66 based on Ce and Zr, applied amine functionalization, and incorporated it into a PEBAX matrix. The resulting MMMs showed a CO_2_/N_2_ selectivity of 76.4 and a CO_2_ permeability of 100.7 Barrer at a low loading of 3 wt.%, together with good pressure resistance, indicating their potential for industrial operation.

Recent work on bimetallic MOFs incorporated into polymer matrices has reported improved gas-separation performance. In this study, three bimetallic MOF-74 materials—Ni-Cu-MOF-74, Ni-Co-MOF-74, and Ni-Zn-MOF-74—are incorporated into a polydimethylsiloxane (PDMS) matrix to prepare mixed matrix membranes (MMMs). Their CO_2_/N_2_ separation performance is examined through single-gas permeation tests. Structural and morphological features of the membranes are analyzed using SEM, XRD, and FT-IR.

## 2. Materials and Methods

### 2.1. Materials Used

The following materials were used for the synthesis of mixed matrix membranes (MMMs):

- Polydimethylsiloxane (PDMS, CAS No. 63148-62-9) obtained from Sigma Aldrich, St. Louis, MO, USA;
- Toluene (CAS No. 208-88-3, Mw = 92.14 g mol^−1^, ACS reagent grade, 99.7% purity) purchased from Sigma Aldrich, St. Louis, MO, USA;
- Ni-Cu-MOF-74, Ni-Co-MOF-74, and Ni-Zn-MOF-74 fillers obtained from another Research group within the institute;
- High-purity CO_2_ (99.999 vol%) and N_2_ (99.999 vol%) gases purchased from Paradise Gases, Islamabad, Pakistan.

All chemicals, solvents, and reagents were used as received without additional purification.

### 2.2. Synthesis of Metal–Organic Frameworks (MOFs)

Ni-M-MOF-74 (M = Cu, Co, Zn) fillers were prepared by a solvothermal route, following the method of Asad et al. [37], with minor adjustments to the metal composition. In a typical synthesis, 1 mmol of 2,5-dihydroxyterephthalic acid (H_4_DOBDC) was dissolved in a mixed solvent of DMF, ethanol, and deionized water (1:1:1, *v*/*v*/*v*). In separate solutions, Ni(NO_3_)_2_·6H_2_O (99 mol%) and a secondary metal nitrate—Cu(NO_3_)_2_·6H_2_O (2 mol%), Co(NO_3_)_2_·6H_2_O (1 mol%), or Zn(NO_3_)_2_·6H_2_O (1 mol%)—were dissolved in water and then combined with the ligand solution under stirring. The resulting mixtures were placed in Teflon-lined stainless-steel autoclaves and heated at 120 °C for 12 h. After cooling to room temperature, the solids were collected by centrifugation, washed several times with ethanol and deionized water, and finally dried under vacuum at 60 °C for 24 h to yield Ni-Cu-MOF-74, Ni-Co-MOF-74, and Ni-Zn-MOF-74 powders.

### 2.3. Synthesis of Mixed Matrix Membranes (MMMs)

**Figure 1:**
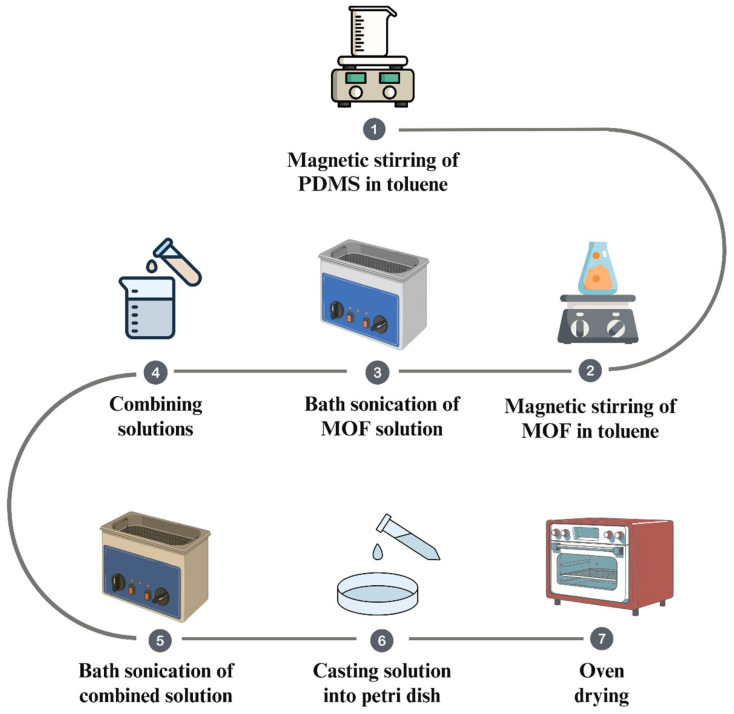
Synthesis procedure for Ni-Cu-MOF-74/PDMS, Ni-Co-MOF-74/PDMS, and Ni-Zn-MOF-74/PDMS MMMs.

The PDMS-based mixed matrix membranes were prepared as follows. First, 4 g of PDMS elastomer (40% *w*/*v*) was dissolved in 10 mL of toluene, and the mixture was stirred magnetically at 300 rpm for 2 h. In parallel, bimetallic MOF particles were dispersed in toluene at a filler loading of 1 wt.% and treated by magnetic stirring, followed by bath sonication, to improve dispersion. The MOF dispersion was then added to the PDMS solution, and the mixture was sonicated for an additional 1 h. To eliminate trapped air, the resulting polymer–filler suspension was degassed under ultrasonic irradiation for 30 min. Finally, the mixture was cast into a Petri dish and dried in an oven at 80 °C for 2–3 h to obtain a dense membrane with a thickness in the range of 170–180 µm. The synthesis procedure is outlined in Figure 1.

## 3. Testing and Characterization

### 3.1. Fourier Transform Infrared (FT-IR) Spectroscopy

FT-IR spectroscopy was used to examine the chemical structure of mixed-matrix membranes (MMMs) containing bimetallic MOFs dispersed in a polydimethylsiloxane (PDMS) matrix. For comparison, FT-IR spectra of the corresponding bimetallic MOF powders were also recorded. Measurements were carried out on a Perkin-Elmer Spectrum 100 FT-IR spectrometer over the wavenumber range 4000–400 cm^−1^ with a resolution of 4 cm^−1^.

### 3.2. Scanning Electron Microscopy

SEM imaging was carried out using a JEOL JSM-6490LA (JEOL Ltd., Tokyo, Japan) analytical low-vacuum instrument. For the membranes, both the surface and cross-section were examined. Cross-sectional images were recorded at magnifications between 250× and 5000×, and surface images between 500× and 10,000×. For the MOF particles, only surface morphology was analyzed at magnifications ranging from 250× to 10,000×. These measurements were used to assess the morphology and internal structure of the MMMs and the MOF fillers.

### 3.3. X-Ray Diffraction

XRD measurements were used to examine the crystallographic structure of the MOF filler particles and the PDMS-based MMMs, and to assess how incorporation into the polymer matrix affected MOF crystallinity. The patterns were recorded on a Bruker D2 (Bruker AXS GmbH, Karlsruhe, Germany) Phaser diffractometer equipped with a copper sample holder, using Cu Kα radiation (λ = 1.5406 Å) at 30 kV and 10 mA.

### 3.4. Brunauer–Emmett–Teller (BET)

The specific surface area and porosity of the membranes were obtained from nitrogen adsorption isotherms using the BET method on an ASAP 2460 Surface area and Porosimetry Analyzer (Micromeritics Instrument Corporation, Norcross, GA, USA). These measurements provide quantitative information on the accessible surface and pore structure, parameters that directly influence gas uptake and diffusion and are therefore important for interpreting the membranes’ separation performance.

### 3.5. Gas Permeation

Gas permeation experiments aim to provide a method for calculating selectivity and permeability, as these factors significantly influence the performance of a given membrane [38]. The gas flow rate, membrane area, membrane thickness, and pressure differential across the membrane may all be used to calculate a gas’s permeability. It is depicted in mathematical form in Equation (1) as shown [39]:

P_A_ = (Q × ∆l)/(A × ∆P) (1)

Here, P_A_ is the gas A permeability through the membrane (expressed in cm^3^-cm/s-cm^2^-cm Hg), Q is the flow rate (volumetric) of the gas (expressed in cm^3^/s), Δl is the thickness of the membrane (expressed in cm), and ΔP is the pressure difference.

Selectivity, defined as the ratio of permeabilities of two gases, is represented mathematically in Equation (2) [40]:

α_A/B_ = P_A_/P_B_ (2)

where α_A/B_ is the selectivity of gas A over gas B.

**Figure 2:**
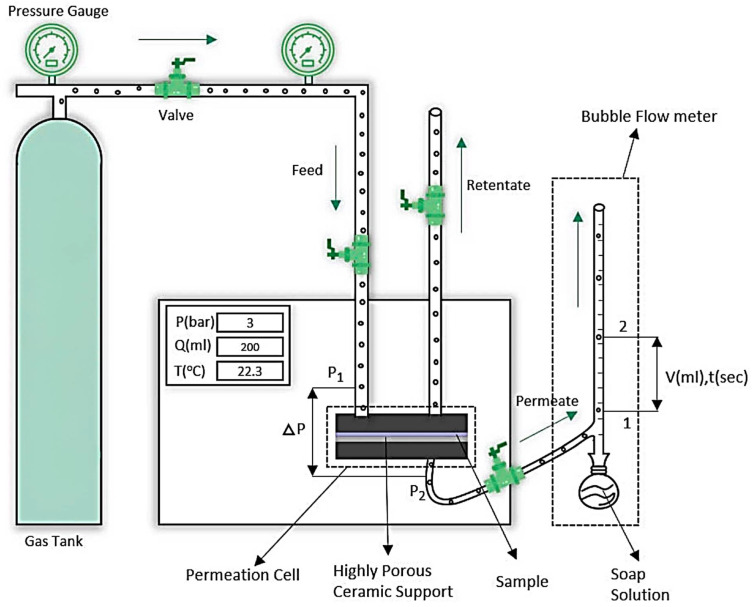
Schematic representation of the gas permeability test system [41].

Single gas permeation testing was conducted using a standard Gas Permeability Test System from PHILOS, (Gwangmyeong-si, South Korea). The system features a circular stainless steel permeation rig with an active surface area of 8 cm^2^. The test membrane sample was secured between two porous ceramic plates, and rubber O-rings prevented leakage. Carbon dioxide and nitrogen gases were used as test gases, and experiments were performed at pressures ranging from 2 to 5 bars, with readings taken at 1-bar increments. The gas permeation rate was measured using a bubble flow meter connected to the permeate side of the system. All tests were conducted at room temperature (25 °C; 298 K), and each membrane was tested three times under identical conditions to verify reproducibility. These parameters were used to calculate the permeability of CO_2_ and N_2_ gases using Equation (1). Permeation testing was also conducted for PDMS-based MMMs containing 1 wt.% Ni-Cu-MOF-74, Ni-Co-MOF-74, and Ni-Zn-MOF-74 particles. The experimental setup is schematically represented in Figure 2 [41].

## 4. Analysis

### 4.1. Fourier Transform Infra-Red (FT-IR) Spectroscopic Analysis

**Figure 3:**
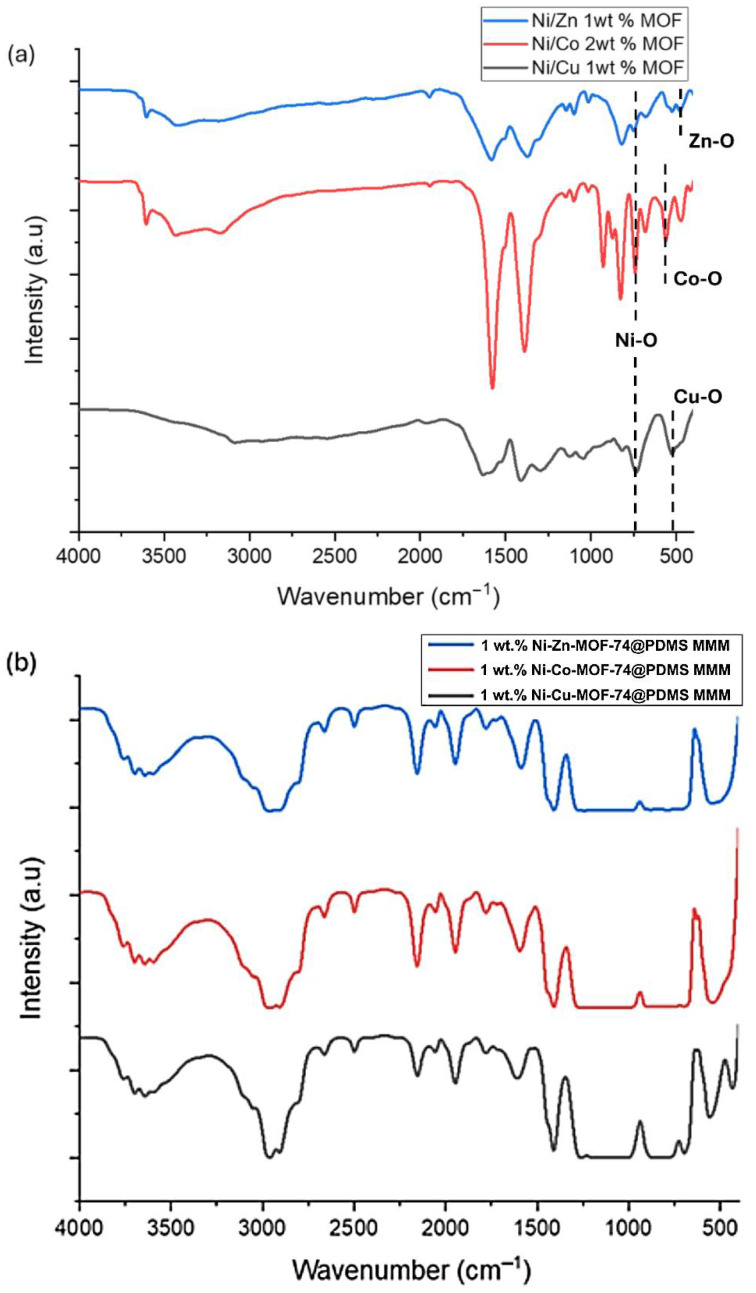
(**a**) FT-IR spectrum for Ni-Cu-MOF-74, Ni-Co-MOF-74, and Ni-Zn-MOF-74; (**b**) FT-IR spectra for MOF-based MMMs for all MOF samples.

Three types of MOF particles—Ni-Co-MOF-74, Ni-Zn-MOF-74, and Ni-Cu-MOF-74—were examined, together with the corresponding MMMs containing 1 wt.% of each filler. The FT-IR spectra of the MOFs and MMMs are shown in Figure 3. The MOF powders displayed the expected characteristic absorption bands associated with their framework functional groups. Similar bands were observed in the spectra of the MMMs, confirming that the MOF particles were successfully incorporated into the PDMS matrix. The preservation of these bands in the MMMs indicates that the MOF structures remained chemically intact during membrane fabrication. Overall, the FT-IR results clarify the chemical composition of the MMMs and support the interpretation of their behaviour in subsequent gas separation measurements.

#### 4.1.1. Analysis of MOF Particles

Figure 3a represents the FT-IR spectra of the three MOF samples:

***Ni-Cu MOF-74:***

Symmetric and asymmetric stretching vibrations of the carboxylate (–COO^−^) groups are observed at 1630.47 cm^−1^ and 1410.12 cm^−1^, respectively. Bands at 1126.05 cm^−1^ and 817.74 cm^−1^ are assigned to C-H bending vibrations. A strong band at 734.72 cm^−1^ corresponds to Ni-O stretching, while the peak at 525.58 cm^−1^ is attributed to Cu-O stretching vibrations.

***Ni-Co-MOF-74:***

The FT-IR spectrum of Ni-Co-MOF-74 shows bands similar to those of Ni-Cu-MOF-74, with slight shifts, indicating a comparable chemical structure. The bands at 1576.67 cm^−1^ and 1389.06 cm^−1^ are assigned to the asymmetric and symmetric stretching vibrations of the carboxylate groups, respectively. Peaks at 1148.19 cm^−1^ and 826.48 cm^−1^ correspond to C-H bending vibrations. The band at 741.24 cm^−1^ is attributed to Ni-O stretching, while the peak at 560.79 cm^−1^ is assigned to Co-O stretching vibrations.

***Ni-Zn-MOF-74:***

Bands at 1582.06 cm^−1^ and 1371.67 cm^−1^ are assigned to the asymmetric and symmetric stretching vibrations of the carboxylate groups, respectively. Peaks at 1145.05 cm^−1^ and 819.59 cm^−1^ correspond to C-H bending vibrations. The band at 749.27 cm^−1^ is attributed to Ni-O stretching, while the peak at 478.83 cm^−1^ arises from Zn-O stretching and is consistent with a highly crystalline framework. Overall, the FT-IR spectra confirm the presence of 2,5-dihydroxyterephthalate (DOBDC) linkers and metal–oxygen bonds in the MOF structures, indicating successful synthesis and good crystallinity.

#### 4.1.2. Analysis of MOF-Based MMMs

Figure 3b shows the FT-IR spectra of the MMMs containing Ni-Cu-MOF-74, Ni-Co-MOF-74, and Ni-Zn-MOF-74. The spectra include the characteristic bands of the MOFs together with additional signals from the PDMS matrix. Peaks at around 1600 cm^−1^, 1400 cm^−1^, 700 cm^−1^, and 500 cm^−1^ coincide with those observed for the corresponding MOF powders (Figure 3a). These peaks are assigned to the symmetric and asymmetric stretching of the carboxylate groups, C-H bending, and metal–oxygen (Ni-O, Cu-O, Co-O, Zn-O) stretching vibrations.

In addition, intense bands at 805 cm^−1^, 1024 cm^−1^, and 1258 cm^−1^ correspond to Si-C-H, Si-O-Si, and Si-C vibrations of the PDMS phase, respectively. A weak band near 2960 cm^−1^ is attributed to sp^3^ C-H stretching. The simultaneous presence of MOF-related and PDMS-related bands confirms that Ni-Cu-MOF-74, Ni-Co-MOF-74, and Ni-Zn-MOF-74 were successfully incorporated into the PDMS matrix and that the hybrid MMMs were properly formed.

### 4.2. SEM Analysis

**Figure 4:**
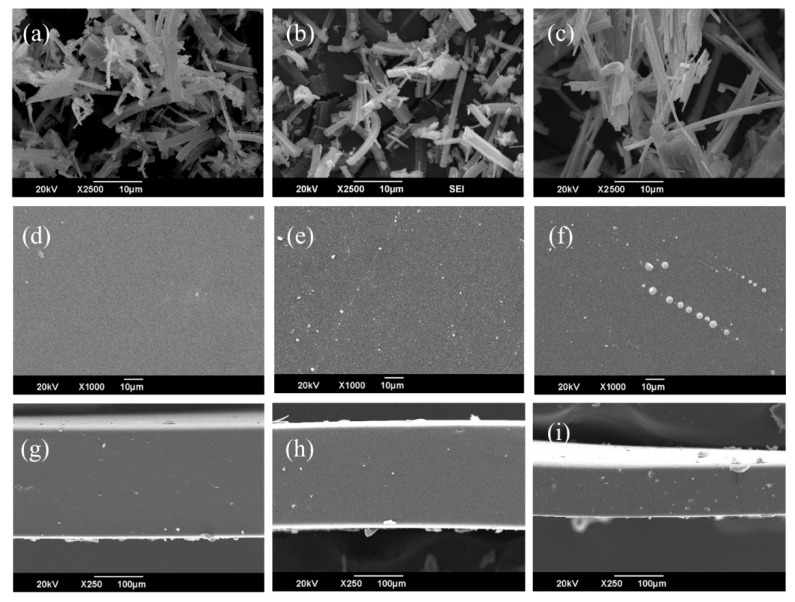
SEM micrographs of (**a**) Ni-Cu-MOF-74, (**b**) Ni-Co-MOF-74, and (**c**) Ni-Zn-MOF-74; surface morphology of MMMs based on (**d**) Ni-Cu-MOF-74, (**e**) Ni-Co-MOF-74, and (**f**) Ni-Zn-MOF-74; and cross-sectional morphology of MMMs based on (**g**) Ni-Cu-MOF-74, (**h**) Ni-Co-MOF-74, and (**i**) Ni-Zn-MOF-74.

SEM analysis was used to examine the morphology of Ni-Cu-MOF-74, Ni-Co-MOF-74, and Ni-Zn-MOF-74 particles and of the corresponding MMMs containing 1 wt.% of each filler in the PDMS matrix. Representative micrographs are shown in Figure 4. The images of the MOF powders (Figure 4a–c) indicate uniform particle morphology, in good agreement with previous reports [42,43]. The surface SEM images of the MMMs (Figure 4d–f) show dense, continuous membranes without visible cracks or voids. The MOF particles appear well dispersed within the PDMS phase, with no obvious agglomeration, suggesting good compatibility and adhesion between the fillers and the polymer [44]. This uniform dispersion is important for preserving both selectivity and permeability in gas separation [45]. Cross-sectional images (Figure 4g–i) reveal compact membrane structures with an even distribution of MOF particles across the membrane thickness and no evidence of interfacial voids or phase separation. These observations further support the presence of strong interfacial bonding between the MOF fillers and the PDMS matrix.

### 4.3. X-Ray Diffraction (XRD) Analysis

**Figure 5:**
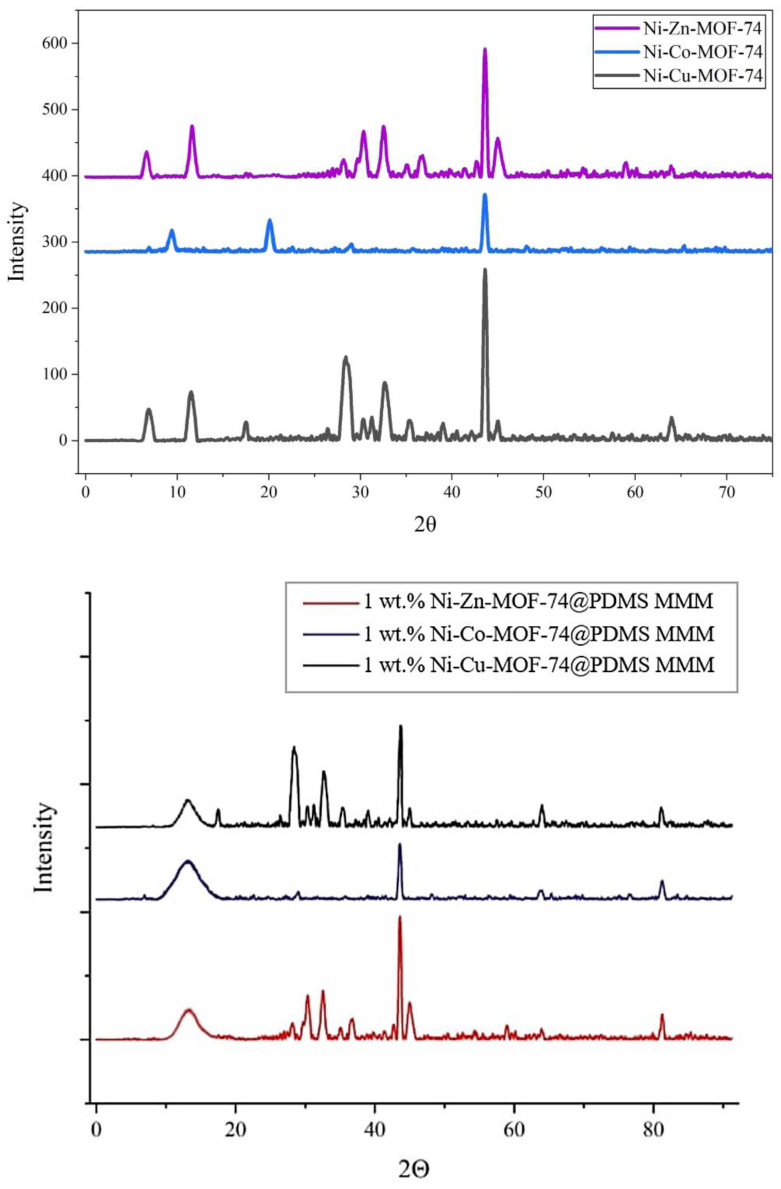
XRD spectra for Ni-Cu-MOF-74, Ni-Co-MOF-74, and Ni-Zn-MOF-74 samples.

XRD analyses were carried out for Ni-Cu-MOF-74, Ni-Co-MOF-74, and Ni-Zn-MOF-74 samples. The results of the analysis are represented in Figure 5 as shown:

#### 4.3.1. XRD Analysis of MOF Samples

The XRD patterns of Ni-Cu-MOF-74, Ni-Zn-MOF-74, and Ni-Co-MOF-74 exhibit the expected reflections corresponding to their crystalline frameworks. For Ni-Cu-MOF-74, a sharp reflection at 2θ = 43.2° is assigned to the Ni-based structure [37], while peaks at 6.7° and 11.6° correspond to the Cu-containing framework and agree with reported data [46]. In the case of Ni-Zn-MOF-74, the pattern includes the Ni-related peak at 43.2° together with reflections at 6.7° and 11.7° attributed to the Zn-based structure, consistent with previous studies [37,47]. For Ni-Co-MOF-74, a peak at 43.2° again indicates the Ni-based structure, and additional reflections at 9.2° and 20.2° are associated with the Co-containing framework [37,48]. Overall, the XRD profiles of the three samples are very similar, indicating that they share the same underlying topology [49]. The results, therefore, support the formation of isostructural MOFs in which the metal centres are varied while the framework structure is preserved [49].

#### 4.3.2. XRD Analysis of MOF-Based MMMs

X-ray diffraction (XRD) was used to study the structure of PDMS-based mixed matrix membranes containing Ni-Cu-MOF-74, Ni-Zn-MOF-74, and Ni-Co-MOF-74 at a loading of 1 wt.% relative to PDMS. The diffraction patterns of the membranes show the characteristic MOF peaks superimposed on the broad amorphous halo of the PDMS phase, confirming that the crystalline fillers were successfully incorporated into the polymer matrix. The MOFs retain their signature reflections at higher 2θ values, corresponding to their well-defined crystalline planes and consistent with the reported structures of the respective frameworks. The persistence of these peaks after incorporation indicates that the MOF crystallinity is preserved within the PDMS matrix. Thus, the membranes consist of an amorphous PDMS phase containing dispersed crystalline MOF domains. This combination is expected to favour gas separation by coupling the molecular sieving behaviour of the MOFs with the intrinsic permeation characteristics of PDMS, making these MMMs suitable candidates for CO_2_ capture applications.

### 4.4. Gas Separation Performance Analysis

**Figure 6:**
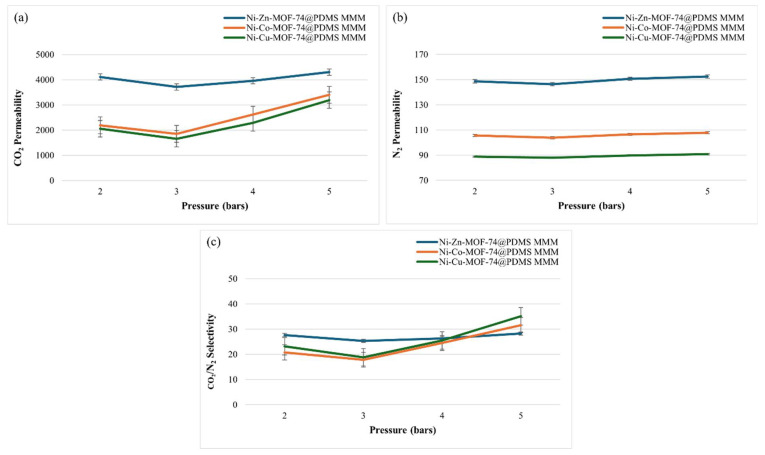
Graphical representation of (**a**) CO_2_ permeability, (**b**) N_2_ permeability (**c**) CO_2_/N_2_ selectivity for MOF-incorporated MMMs.

The gas separation performance of the MOF-based MMMs was evaluated by single-gas permeation experiments with CO_2_ and N_2_ using a stainless-steel permeation cell. The permeation data for membranes containing Ni-Cu-MOF-74, Ni-Co-MOF-74, and Ni-Zn-MOF-74 are listed in Table 1. The corresponding trends in CO_2_ and N_2_ permeability and CO_2_/N_2_ selectivity as a function of feed pressure are shown in Figure 6.

In Figure 6a, the CO_2_ permeability of the MMMs containing Ni-Cu-MOF-74, Ni-Co-MOF-74, and Ni-Zn-MOF-74 generally increases with feed pressure, with a small decrease at 3 bar. This deviation is consistent with swelling and sorption effects in the PDMS phase, which can temporarily hinder gas transport [50]. Among the samples, the Ni-Zn-MOF-74-based MMM shows the highest CO_2_ permeability. This behaviour is associated with weaker framework–gas interactions and the slightly enlarged pore structure induced by Zn^2+^ incorporation, which reduces metal-ligand bond strength and promotes faster gas diffusion through the membrane [51,52,53]. The N_2_ permeability data in Figure 6b follow a similar pressure dependence, including a dip at 3 bar, and again the Ni-Zn-MOF-74 MMM exhibits the highest values, consistent with its larger effective pore size. The resulting CO_2_/N_2_ selectivities are plotted in Figure 6c. The 1 wt.% Ni-Cu-MOF-74@PDMS membrane achieves the highest selectivity, with a value of 35.10. Favourable interfacial interactions between the MOF fillers and the PDMS phase lead to a dense, defect-free morphology in the Ni-Cu-MOF-74-based MMM [54]. This good contact at the filler–polymer interface promotes more effective, selective transport pathways for CO_2_, as reflected in the high separation performance. By contrast, the Ni-Zn-MOF-74 MMM, although showing the highest CO_2_ permeability, exhibits the lowest CO_2_/N_2_ selectivity, illustrating the typical permeability–selectivity compromise observed in polymer-based MMMs [55]. Considering both parameters, the Ni-Cu-MOF-74@PDMS membrane offers the most favourable combination, with a CO_2_ permeability of 3188.25 Barrer and a CO_2_/N_2_ selectivity of 35.10, and is therefore identified as the best-performing composition in this work.

### 4.5. BET Analysis

The physical properties of the nickel-based MMMs containing Ni-Cu-MOF-74, Ni-Zn-MOF-74, and Ni-Co-MOF-74 are listed in Table 2. BET measurements show surface areas of 0.1166 m^2^/g for the Ni-Cu-MOF-74 MMM, 0.0070 m^2^/g for the Ni-Zn-MOF-74 MMM, and 0.0666 m^2^/g for the Ni-Co-MOF-74 MMM. Among the three, the Ni-Cu-MOF-74 membrane has the largest surface area, consistent with its higher CO_2_ permeability relative to pristine PDMS. The increased BET surface area indicates a more developed porous structure, providing a greater number of accessible sites for gas adsorption and transport.

The BET and permeation data indicate that the Ni-Cu-MOF-74-based MMM combines high surface area with strong framework–CO_2_ interactions. The presence of both Ni and Cu metal centres in the MOF lattice likely increases the number and variety of CO_2_ adsorption sites, enhancing CO_2_ affinity. This bimetallic composition also improves the dispersion of the MOF particles within the PDMS phase, limiting interfacial defects and strengthening polymer–filler contact. The higher BET surface area of the Ni-Cu-MOF-74 membrane is consistent with a more accessible porous network, which promotes CO_2_ uptake and diffusion through the matrix. These structural features account for the observed increase in CO_2_ permeability and CO_2_/N_2_ selectivity, making the Ni-Cu-MOF-74-based MMM well suited for CO_2_ separation applications.

## 5. Performance Comparison

**Figure 7:**
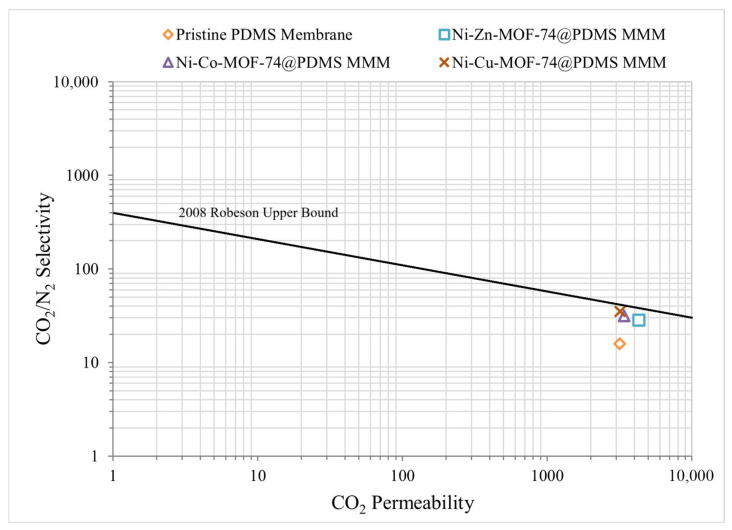
Performance Analysis of Membrane Samples.

The Robeson (2008) [16] upper bound was used as a reference to assess the balance between gas permeability and ideal selectivity. The performance of all fabricated membranes plotted in Figure 7 shows that the MOF-based MMMs shift above the position of pristine PDMS, with simultaneous increases in CO_2_ permeability and CO_2_/N_2_ selectivity. Among the modified membranes, the PDMS membrane containing 1 wt.% Ni-Cu-MOF-74 exhibits the largest deviation from the upper bound of pristine PDMS, reflecting the most pronounced improvement in separation performance. The PDMS/Ni-Zn-MOF-74 and PDMS/Ni-Co-MOF-74 MMMs also show clear gains in both permeability and selectivity relative to the unfilled polymer. These trends indicate that incorporation of MOF-74-type fillers effectively enhances the transport properties of the PDMS matrix and yields a more favourable compromise between CO_2_ permeability and CO_2_/N_2_ selectivity.

## 6. Conclusions

Membrane-based carbon capture offers a practical route to reducing CO_2_ emissions from industrial sources. In this work, mixed matrix membranes (MMMs) were prepared using PDMS as the continuous phase and Ni-Cu-MOF-74, Ni-Co-MOF-74, or Ni-Zn-MOF-74 as dispersed fillers. The membranes were fabricated by solution casting and examined by XRD, SEM, FT-IR, and BET analysis. These characterizations confirmed the formation of smooth, dense, defect-free films with well-dispersed MOF particles in the PDMS matrix. Single-gas permeation tests with CO_2_ and N_2_ showed that incorporation of Ni-Cu-MOF-74 at 1 wt.% provided the most favourable transport properties. The 1 wt.% Ni-Cu-MOF-74@PDMS membrane reached a CO_2_/N_2_ selectivity of 35.10 and a CO_2_ permeability of 3188.25 Barrer, both higher than those of pristine PDMS, and representing a good compromise between permeability and selectivity. Overall, the results indicate that PDMS-based MMMs containing bimetallic MOF-74 fillers, particularly Ni-Cu-MOF-74, are suitable candidates for CO_2_/N_2_ separation in industrial gas treatment and CO_2_ capture processes.

## Figures and Tables

**Table 1:** Gas permeation analysis.

	Pristine PDMS Membrane	1 wt.% Ni-Zn-MOF-74@PDMS MMM	1 wt.% Ni-Cu-MOF-74@PDMS MMM	1 wt.% Ni-Co-MOF-74@PDMS MMM
Maximum CO_2_ Permeability (Barrer)	3176.05 ± 0.10	4303.27 ± 0.11	3188.25 ± 0.13	3406.09 ± 0.11
Maximum N_2_ Permeability (Barrer)	200.54 ± 0.12	152.43 ± 0.07	90.83 ± 0.09	107.91 ± 0.10
Optimum CO_2_/N_2_ Selectivity	15.84	28.23	35.10	31.56

**Table 2:** BET Analysis.

Adsorbent Materials	Surface Area (m^2^/g)
1 wt.% Ni-Cu-MOF-74@PDMS MMM	0.1166 m^2^/g
1 wt.% Ni-Zn-MOF-74@PDMS MMM	0.0070 m^2^/g
1 wt.% Ni-Co-MOF-74@PDMS MMM	0.0666 m^2^/g

## Data Availability

The original contributions presented in this study are included in the article. Further inquiries can be directed to the corresponding author.
